# Supplementary material for: Evaluation of Aciclovir-Induced Nephrotoxicity in Critically Ill Patients: A Propensity-Matched Cohort Study
Source: J Clin Med. 2025 Feb 20;14(5):1409. doi: 10.3390/jcm14051409 (PMC11900339; doi:10.3390/jcm14051409)
Supplement: Supplementary file 1 [file jcm-14-01409-s001.zip › jcm-3439232-supplementary.pdf]

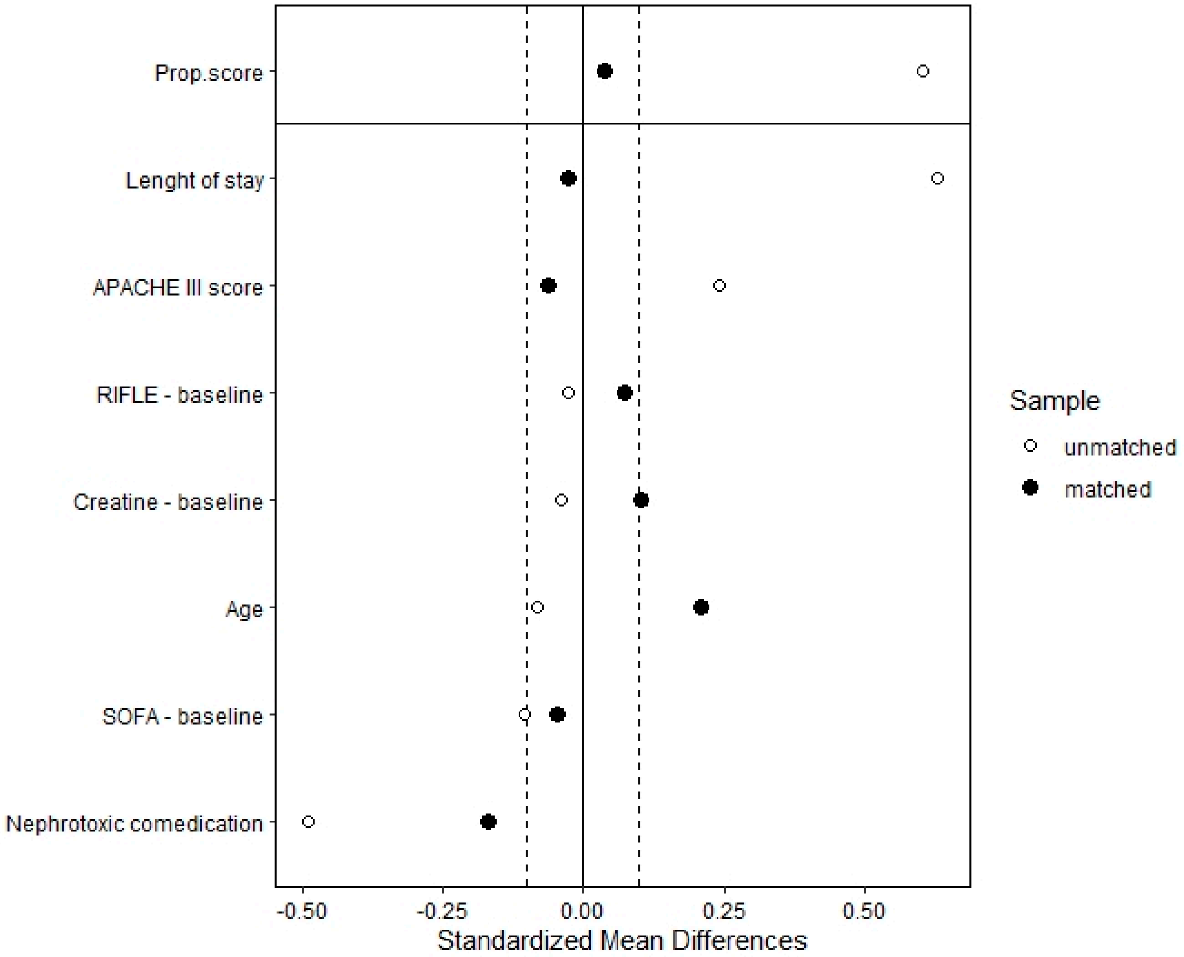

Figure S1. Love plot of the standardized mean differences of the covariates before and after propensity score matching. The dashed lines represent the -10% and +10% threshold for the absulte standardized mean difference.
